# First Serological Evidence of Crimean-Congo Hemorrhagic Fever Virus Infections in Croatia: A Multispecies Surveillance Approach Emphasising the Role of Sentinel Hosts

**DOI:** 10.3390/v17101335

**Published:** 2025-09-30

**Authors:** Gorana Miletic, Ivona Coric, Snjezana Kovac, Alenka Skrinjaric, Magda Kamber Taslaman, Margarita Bozikovic, Ljubo Barbic, Viktor Masovic, Jelena Prpic, Lorena Jemersic, Vladimir Stevanovic

**Affiliations:** 1 Department of Microbiology and Infectious Diseases with Clinic, Faculty of Veterinary Medicine, University of Zagreb, 10000 Zagreb, Croatia; icoric@vef.unizg.hr (I.C.); skovac@vef.unizg.hr (S.K.); askrinjaric@vef.unizg.hr (A.S.); ljbarbic@vef.unizg.hr (L.B.); 2 Laboratory for Classical Swine Fever Diagnosis, Molecular Virology and Genetics, Virology Department, Croatian Veterinary Institute, 10000 Zagreb, Croatia; kamber@veinst.hr (M.K.T.); bozikovic@veinst.hr (M.B.); balatinec@veinst.hr (J.P.); jemersic@veinst.hr (L.J.); 3 Poultry Center, Croatian Veterinary Institute, 10000 Zagreb, Croatia; masovic@veinst.hr

**Keywords:** CCHFV, seroprevalence, vector-borne disease, sentinel animals, Croatia

## Abstract

Crimean-Congo hemorrhagic fever virus (CCHFV) is a tick-borne zoonotic pathogen of growing public health concern in southeastern Europe. This study provides the first serological evidence of CCHFV circulation in Croatia, based on testing 1473 serum samples from farm and companion animals, including sheep, horses, cattle, goats, dogs, and cats. A total of 109 samples (7.4%) tested positive for CCHFV antibodies using a commercially available enzyme-linked immunosorbent assay (ELISA) kit. The highest seroprevalence was recorded in sheep (28.3%), followed by horses (4.3%) and a single cat (0.5%), with no antibodies detected in cattle, goats, or dogs. Almost all seropositive animals originated from coastal and subcoastal Croatia, where *Hyalomma* ticks are present. Only two seropositive cases were detected in continental areas. Sheep samples from several farms in Zadar County showed intra-farm seropositivity rates of up to 85.7%, suggesting localised virus circulation likely influenced by vector distribution and farm-level practices. No viral ribonucleic acid (RNA) was detected by quantitative reverse transcription polymerase chain reaction (qRT-PCR), consistent with the transient nature of viremia in most animal hosts. These findings confirm the silent circulation of CCHFV in Croatia and reinforce the need for targeted, regionally adapted surveillance strategies that integrate multiple hosts and support early warning systems aligned with the One Health concept.

## 1. Introduction

Crimean-Congo hemorrhagic fever virus (CCHFV) is an RNA virus classified in the *Orthonairovirus haemorrhagiae* species, genus *Orthonairovirus,* a member of the family *Nairoviridae* [1]. It is a pathogen that poses a significant threat to public health, with a human case fatality rate of up to 30% [2]. The virus was first identified during World War II in the Crimean region. It was later found to be antigenically similar to a virus isolated in 1956 in what is now the Democratic Republic of Congo. This led to the naming of the virus as Crimean-Congo hemorrhagic fever virus [2,3]. Since its discovery, CCHFV has been reported in countries across Africa, Asia, Eastern Europe, and the Mediterranean basin [4,5,6].

The distribution of the virus closely correlates with the presence of ticks from the genus *Hyalomma*, which are considered the primary vector and potential reservoir of CCHFV [7]. The virus replicates in a variety of mammalian and avian hosts in endemic regions. Domestic animal hosts typically include small ruminants; however, serological evidence of infection has also been detected in equids, cattle, and wild animals [8,9,10].

Humans and laboratory mice are the only species that develop clinical disease following infection [11]. The initial symptoms in humans are nonspecific, resembling a common cold. This early phase is particularly critical for human-to-human transmission, making Crimean-Congo hemorrhagic fever (CCHF) a significant nosocomial threat in endemic areas [12]. A considerable proportion of confirmed human cases is associated with occupational exposure, especially among individuals working on farms or in slaughterhouses, with cattle frequently implicated [13,14]. Infected animals, although asymptomatic, undergo a brief viremic phase during which their body fluids pose a substantial risk for zoonotic transmission [11]. The risk is heightened by the fact that infection in animals often goes unnoticed. Consequently, detection of infection in animals usually occurs during outbreak investigations in humans or through targeted research, primarily via serological testing for virus-specific antibodies. The subclinical nature of the infection and the short duration of the viremic period, combined with close human-to-animal contact in high-risk settings, make early detection challenging and limit the effectiveness of outbreak prevention [8]. Considering these risks, there is a clear need for improved surveillance systems in risk areas, particularly those that incorporate more animal species that could serve as sentinels during the early stages of virus circulation.

Certain regions of Croatia, due to the abundance of vectors and potential reservoir hosts, are at considerable risk for disease emergence. In response to the slow yet steady spread of the virus across the Balkan Peninsula, such as the appearance in bordering Bosnia and Herzegovina in 2022 [15], the Croatian public health community has remained aware of the potential introduction of CCHFV within national borders [4,7,16,17]. As a proactive step, a surveillance study was launched to assess viral activity in multiple domestic and companion animal species.

## 2. Materials and Methods

For this study, Croatia was divided into four broader regions based on geographical position and the documented presence of the *Hyalomma marginatum* tick species [18]: Littoral Croatia and Dalmatia, where *Hyalomma* ticks are present, and Eastern and Central Croatia, where their presence has not been documented to date. Initially, we tested 304 horse, 304 dog, and 196 cat serum samples, along with 276 samples from cattle and 57 from goats. For most samples, data on species, sex, age, and geographical location were available (Table 1). These samples were collected during 2024 as part of the CROOH project (“Establishment of a coordinated disease surveillance system in Croatia in accordance with the One Health approach”, Project No. 101132755).

**Figure 1:**
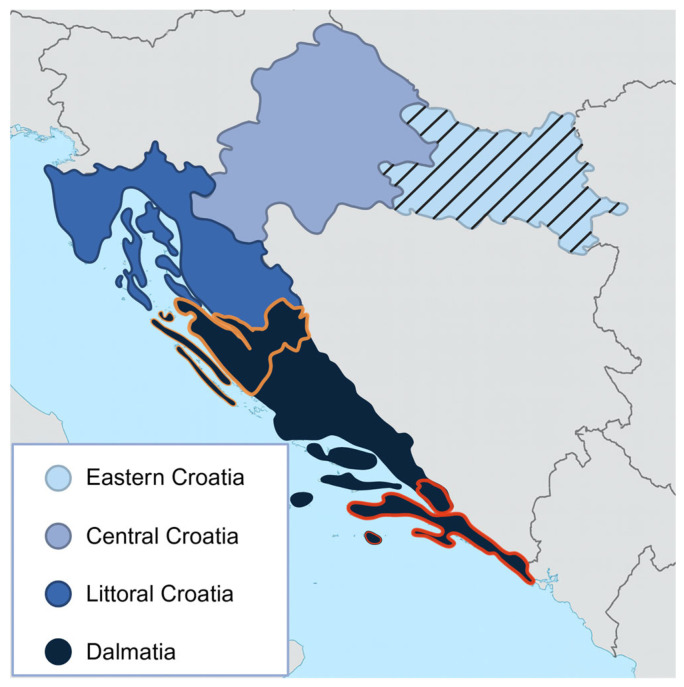
The map of Croatia is divided into four regions representing the sampling areas. Horse, dog, and cat sera were collected across all four regions. Cattle and goat sera were obtained exclusively from Eastern Croatia (indicated with stripes), while sheep sera were collected from Zadar County (outlined in orange) and Dubrovnik–Neretva County (outlined in red).

In the second phase of the study, 184 archived sheep serum samples collected in one county in Dalmatia (Zadar County) in 2024 and 152 collected in Dubrovnik-Neretva County in Dalmatia in 2023 were tested. Sample size calculations for the first round of testing were performed using the RiBESS software version 0.1.2 [19], based on an expected seroprevalence of 30% in domestic ruminants and 10% in horses and pet animals, as sentinel species, based on previously reported seroprevalences in neighbouring countries and studies in respective sentinel animals [8,20,21]. Archived sheep samples originated from previous diagnostic and surveillance activities. Their numbers were determined by availability rather than sample size calculation. Archived sheep samples were limited to the central and south coastal counties of Zadar and Dubrovnik in the Dalmatia region (Figure 1).

Since all samples used in this research were remnant serum samples collected by veterinarians during routine diagnostics and national surveillance for other diseases, not specifically for this study, no animal study permit was required.

All samples were stored at −80 °C until testing.

Serum samples were tested for anti-CCHFV nucleoprotein antibodies using the ID Screen^®^ CCHF Double Antigen Multi-species ELISA kit (IDvet, Grabels, France), according to the manufacturer’s instructions [22,23]. This ELISA employs a double-antigen sandwich (DAS) format, allowing for the detection of total antibodies (IgG, IgM, and IgA). It does not rely on species-specific secondary antibodies, which makes it convenient for multispecies testing. According to the manufacturer, the assay has a specificity of 100% and a sensitivity of 98.9% across multiple animal species, with no cross-reactivity with related nairoviruses, including Hazara virus, Dugbe virus, and Nairobi Sheep Disease Virus. However, due to antigenic similarity, cross-reactivity with Aigai virus cannot be ruled out. Optical density (OD) values were read at 450 nm using a TECAN Sunrise ELISA microplate reader (Tecan Trading AG, Männedorf, Switzerland). The test was valid if the mean OD value of the positive control was greater than 0.350 and the OD ratio of the positive and negative controls was greater than three. The S/P% ratio was calculated for each sample by dividing the OD of the sample by the OD of the positive control. Samples that had S/P% greater than 30% were considered positive. All samples were tested in duplicate.

Serum samples that tested positive were subsequently tested by quantitative (q) reverse-transcription (RT) PCR. RNA extraction was performed using the IndiMag Pathogen Kit (Indical, Leipzig, Germany), with automated processing carried out on the KingFisher Flex nucleic acid extraction system (Thermo Fisher Scientific, Waltham, MA, USA). Isolates that were not immediately subjected to qPCR were stored at −20 °C until use. Molecular analysis was conducted using a commercial one-step RT-PCR kit, CCHF Virus One-Step RT-qPCR Kit (NZYtech, Lisboa, Portugal) [24]. This assay enables simultaneous reverse transcription and amplification in a single reaction tube, designed to detect a wide range of CCHFV genetic variants with high specificity. According to the manufacturer’s documentation, the test targets conserved regions of the viral genome and has a detection limit of approximately 10 copies per reaction. Real-time PCR reactions were performed using the CFX96 Real-Time PCR Detection System (Bio-Rad, Hercules, CA, USA) according to the manufacturer’s protocol. Samples with a Ct (cycle threshold) value below 40 in the FAM channel were considered positive.

Statistical analyses were performed using Statistica v.14 (TIBCO Software Inc., Palo Alto, CA, USA, 2020) and MedCalc Odds Ratio Calculator v.23 (MedCalc Software Ltd., Ostend, Belgium, 2025). Exact binomial 95% confidence intervals (CIs) were calculated for species-specific prevalence estimates. Descriptive statistics were used to summarise seropositivity by species, age group, sex, and geographical region. Categorical variables were compared using Pearson’s chi-square test or Fisher’s exact test when expected frequencies were low. Odds ratios (ORs) with 95% CIs were calculated to describe associations between potential risk factors and seropositivity. For sheep, logistic regression with age as a continuous variable was applied to assess the effect of age on seropositivity. A significance level of *p* < 0.05 was used.

## 3. Results

### 3.1. Serological Results in Different Animal Species

Out of 1473 serum samples tested, 109 were positive for CCHFV nucleocapsid antibodies. These results represent the first serological evidence of CCHFV infection in Croatia. Among the species tested, the highest seroprevalence was observed in sheep at 28.3% (95%CI 23.5–33.4), followed by 4.3% in horses (95%CI 2.3–7.2), and 0.5% in domestic cats (95%CI 0.01–2.8). All samples collected from cattle (*n* = 276), goats (*n* = 57), and dogs (*n* = 304) tested negative (Table 2).

### 3.2. Geographic Distribution of Seropositive Animals

**Figure 2:**
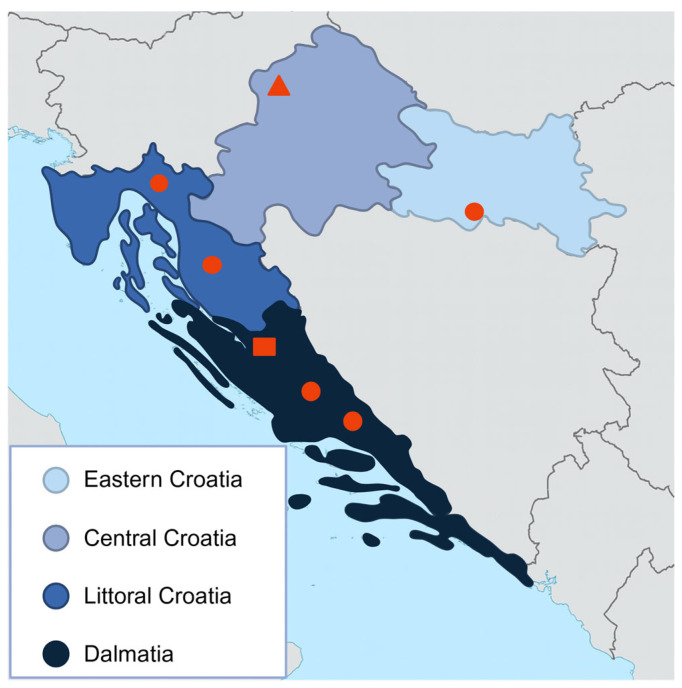
Croatian counties with serologically confirmed CCHFV infection in different animal species. Positive samples are represented as dots for horses, triangles for cats, and squares for sheep.

A total of 788 animals were tested from inland Croatia, and 685 from coastal and subcoastal regions. Only two samples, one feline and one equine, tested positive in the inland region (Eastern Croatia and Central Croatia). On the other hand, 107 samples comprising 95 sheep and 12 horses, tested positive in the coastal/subcoastal region (Littoral Croatia and Dalmatia) (Figure 2).

Out of the 12 seropositive horses located in the coastal and subcoastal regions, eight were found in Littoral Croatia. Positive horses were owned by 11 different individuals, with only two owners having more than one seropositive animal.

**Figure 3:**
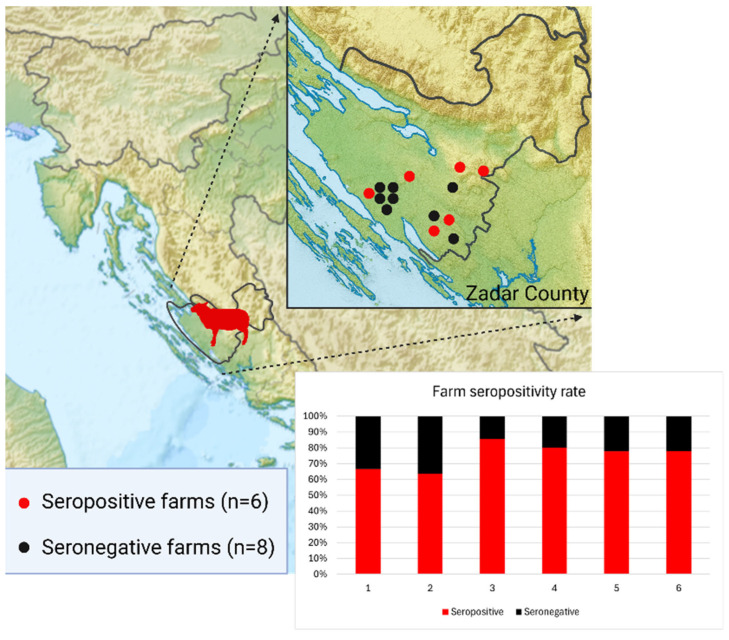
Distribution of seropositive and seronegative sheep farms in Zadar County, with seropositive farms showing uniformly high within-farm seroprevalence.

Notably, all seropositive sheep were sampled in 2024 and originated from one county in Dalmatia (Zadar County). In this county, 184 animals were tested across 14 farms. Eight farms were negative, while six had multiple seropositive cases. Within the positive farms, the intra-farm positivity rate ranged from 63.6% to 85.7% (Figure 3). In contrast, all sheep tested from the Dubrovnik–Neretva County (*n* = 152) in Dalmatia were seronegative.

### 3.3. Association of Age and Sex with Seroprevalence in Sheep and Horses

Due to a small number of positive cats, age-related analysis was performed only for sheep from Zadar County and horses.

Among the 184 sheep samples, 16 were lambs (<1 year), 17 were juveniles (1–2 years), and 151 were adults (>2 years). Seroprevalence rates were 6.3% (1/16; 95% CI: 0.2–30.2%) in lambs, 11.8% (2/17; 95% CI: 1.5–36.4%) in juveniles, and 60.9% (92/151; 95% CI: 52.5–68.9%) in adults. A Chi-square test revealed significantly higher seroprevalence in adult sheep compared to younger animals (χ^2^ = 29.24; *p* < 0.001). When age was considered as a continuous variable, the likelihood of seropositivity increased by approximately 1.35 times for every year of age (OR = 1.35; 95%CI: 1.21–1.53). Information on sex was available for 183 sheep, with six males and 177 females. Among these, one male and 94 females tested positive. Although more females were seropositive, the difference was not significant (*p* = 0.65, Fisher’s exact test).

In horses, age was available for 196 animals, including eight foals (<1 year), 46 juveniles (1–3 years), 104 adults (4–14 years), and 38 seniors (>14 years. Seropositivity was highest among foals (2/8; 25.0%), followed by juveniles (5/46; 10.9%), and lower in adults (4/104; 3.8%) and seniors (1/38; 2.6%). Pairwise comparison using Fisher’s exact test did not reveal statistically significant differences. Information on sex was available for 226 horses: 152 females and 74 males. Among these, seven females and six males tested positive. No significant influence of sex on test outcome was found (OR = 0.55; 95% CI: 0.18–1.69; *p* = 0.362).

### 3.4. Molecular Testing

All seropositive samples were subsequently tested by RT-PCR to determine the presence of CCHFV RNA. All samples tested negative.

## 4. Discussion

In the past decade, CCHFV has continued to spread across the Balkan countries [7]. Although human cases were not confirmed in all countries or areas, CCHFV circulation was reported based on serological screening of animal sera. Romania and Bulgaria regularly report high seroprevalence rates in small ruminants and cattle, even in areas without documented human cases, indicating the silent circulation of the virus [25,26]. Of particular concern for Croatia is Bosnia and Herzegovina, which shares a long natural border. This country reported its first seropositive animals in 2022 through retrospective testing of sheep samples collected in 2018 [15]. Subsequent testing of other domestic species, including cattle, also yielded seropositive results [27], further supporting evidence of undetected viral circulation. In addition, a recent study from Hungary found the highest livestock seroprevalence (1.8%) in the south-central region, close to the Croatian border [28].

Being surrounded by countries with confirmed endemicity and the presence of competent vectors, Croatia was considered a high-risk area for disease emergence. Considering the Hungarian border to the northeast, a targeted serosurvey of sheep in continental Croatia was previously conducted [29]. All tested animals were seronegative; however, they originated from areas where *Hyalomma* ticks have not yet been reported.

This study expanded surveillance to coastal and subcoastal regions of the country, where the presence of *Hyalomma* ticks had already been documented, resulting in the first serological evidence of CCHFV infections in Croatia. The highest seroprevalence was observed in sheep (28.3%, 95% CI 23.5–33.4), followed by horses (4.3%, 95% CI 2.3–7.2), and a single domestic cat (0.5%, 95% CI 0.01–2.8). These results align with trends reported in other endemic countries such as Bulgaria and Turkey, where sheep often show higher seroprevalence rates compared to other livestock species [30,31]. However, unlike those studies, which also documented positive goats and high seroprevalence in cattle, no seropositive cattle or goats were detected here, likely due to the sampling of these species solely from inland regions.

While seroprevalence in horses is less frequently studied, varying rates have been reported from endemic areas, including 16.7% in Benin and as high as 70.3% in Senegal [7,20]. Horses have proven to be highly effective sentinel animals for monitoring CCHFV circulation in regions where the virus is emerging [32], as was the case in our study. Wide distribution, outdoor housing, and documented strong humoral immune responses make them valuable sentinels for early detection of pathogens. Recent immunogenetic research has shown that horses possess unusually high antibody diversity due to their unique immune gene structure, which enables an immune response to low-level and sporadic exposure [33]. This may be important for identifying virus circulation in areas where vector abundance is low, thus providing an early warning system in regions considered at risk but not yet endemic. Unlike livestock species that may serve as amplifying hosts, horses typically develop detectable, long-lasting antibodies without substantial viremia, making them excellent indicators of environmental viral exposure without contributing significantly to onward transmission [34].

Interestingly, the aforementioned Senegalese study also reported a 6.9% seroprevalence in domestic dogs, a species that rarely tests positive for CCHFV and was negative in our survey. Evidence of infection in domestic cats is even scarcer. A recent study from Namibia reported low seroprevalence in cats (1.7%) but a higher rate in dogs (11.5%), attributing this to the free-roaming behaviour of dogs in that setting [21]. In contrast, dog ownership in Croatia is tightly regulated, with few free-roaming animals, which likely limits their exposure to infected ticks. As frequently described in the literature, differences in seroprevalence across species may reflect not only variations in host susceptibility and immune response but also differences in animal management practices, vector exposure, and the regional distribution of competent tick species [35].

The geographic distribution of seropositive cases in this study was markedly uneven. Cattle, sheep, and goat samples were available from limited areas, but surveillance in horses and pet animals involved the whole country. Only two seropositive animals, one horse and one cat, were detected among 788 animals tested in continental Croatia. The strong spatial clustering of cases suggests that the absence of seropositivity in certain areas can be more closely linked to the limited presence of competent tick vectors rather than host species distribution. These isolated findings may represent sporadic transmission events, potentially facilitated by less commonly implicated tick species, such as Rhipicephalus or Dermacentor, which are present in inland regions [18,36]. Alternatively, the undocumented movement of animals from endemic areas could explain these detections. For non-endemic but high-risk countries, surveillance strategies focused on areas with confirmed or ecologically plausible vector presence, rather than merely host density, may provide a more effective framework for early detection and prevention of CCHFV.

Interestingly, significant clustering was observed on a farm level. In Dalmatian Zadar County, six out of fourteen tested sheep farms had high and relatively uniform seropositivity rates, ranging from 63.6% to 85.7%. This finding suggests that, in addition to distribution and clustering of infected vectors, factors such as livestock management practices, pasture maintenance, and overall housing could play a role in facilitating CCHFV transmission. Similar patterns have been reported in other endemic regions. In Afghanistan, studies have highlighted the strong clustering of seropositive animals on specific farms, attributed to the communal use of pastureland, which led to increased tick exposure and virus transmission [37]. These observations underscore the importance of considering not only ecological but also anthropogenic factors when designing surveillance and control strategies for CCHFV. Interestingly, none of the 152 sheep tested from Dubrovnik-Neretva County were seropositive. As the southernmost Dalmatian county, it is likely to have suitable conditions for CCHFV circulation, including the presence of competent tick vectors. Differences in animal husbandry may explain the absence of seropositive results compared to Zadar County, which could include more frequent housing, less use of shared pastures, or generally lower exposure to tick habitats.

Studies in endemic areas consistently found that older animals had significantly higher odds of CCHFV seropositivity, likely due to cumulative exposure to tick-infested pastures [37,38,39]. One study noted that infection rates markedly increased after two years of age, coinciding with when young animals began full-time grazing [40]. Our results in sheep align with these observations, where adults demonstrated markedly higher antibody prevalence compared to younger age groups. Sheep over two years of age were 23.4 times more positive than lambs and 11.7 times more positive than juveniles up to the age of two. This supports the hypothesis that prolonged environmental exposure increases the likelihood of infection. Although seroprevalence in tested horses appeared to be highest in younger age groups, particularly foals and juveniles, statistical analysis did not show significance. This is likely influenced by the small number of seropositive animals (*n* = 13), which limited the power to detect differences between groups.

Although RT-PCR detected no viral RNA in any of the seropositive animals, the serological results provide strong evidence for prior exposure and silent viral circulation in Croatia. Antibody detection, particularly in asymptomatic animals, is a well-established early indicator of virus presence and remains a crucial surveillance tool for pathogens like CCHFV, which cause transient viremia and subclinical infection in most animal hosts. However, in regions where antigenically related nairoviruses (e.g., Aigai virus) co-circulate, like the southern Balkans, some degree of serological cross-reactivity cannot be excluded [41,42]. Although the Aigai virus has not been reported in Croatia or neighbouring countries to date, its circulation in more distant parts of the Balkans suggests that such cross-reactivity remains possible. Additional serological testing, as well as molecular confirmation with follow-up sequencing, would be valuable for differentiating closely related viruses. These findings provide a basis for future targeted vector studies and emphasise the importance of maintaining an integrated, species-diverse surveillance system aligned with the One Health approach.

## 5. Conclusions

This study provides the first serological evidence of CCHFV infection in Croatia. Antibodies were detected in sheep, horses, and a cat, while all tested cattle, goats, and dogs were seronegative. The seroprevalence was highest in sheep, particularly among older individuals. Most seropositive animals were located in coastal and subcoastal regions where the principal vector, *Hyalomma marginatum*, is established. These findings demonstrate the silent circulation of CCHFV among multiple animal species in Croatia, confirming the value of a multispecies surveillance approach. However, molecular confirmation of the virus in animals or vectors is still lacking.

## 6. Research Limitations

This study has several limitations. The distribution of sampled animals varied by species, with cattle and goats originating exclusively from continental regions, and sheep restricted to two coastal counties. This limited our ability to compare seroprevalence across several species and areas. The small number of seropositive animals in some species, particularly cats and horses, restricted the statistical power of analyses between groups, especially when determining the influence of age and sex. The potential co-circulation of some antigenically related nairoviruses, specifically Aigai virus, raises the possibility that detected antibodies may, in part, reflect cross-reactive responses rather than actual CCHFV exposure, notably since molecular confirmation is lacking.

## Figures and Tables

**Table 1:** Sample structure by species, age, sex, and region.

		Age in Years (*n*)					Sex (*n*)			Region (*n*)			
**Species**	**N**	**<1**		**1–2**	**>2**	**NA**	**M**	**F**	**NA**	**EC**	**CC**	**LC**	**D**
**Cattle**	276	-		-	-	276	-	-	276	276	-	-	-
**Goats**	57	-		-	-	57	-	-	57	57	-	-	-
**Sheep**	336	16		17	151	152	6	177	153	-	-	-	336
**Dog**	304	13		71	207	13	125	109	70	128	50	70	56
**Cat**	196	46		37	103	10	78	85	33	101	53	9	33
		**<1**	**1–3**	**4–14**	**>14**	**NA**							
**Horse**	304	8	46	104	38	108	74	152	78	71	58	113	62

Note: NA—not available; EC—Eastern Croatia; CC—Central Croatia; LC—Littoral Croatia; D—Dalmatia.

**Table 2:** Seroprevalence of CCHFV antibodies in different animal species in Croatia.

Species	Tested (*n*)	Positive (*n*)	Prevalence (%)	95%CI (%)
Sheep	336	95	28.3	23.5–33.4
Horses	304	13	4.3	2.3–7.2
Cats	196	1	0.5	0.01–2.8
Cattle	276	0	0.0	0.0–1.3
Goats	57	0	0.0	0.0–6.3
Dogs	304	0	0.0	0.0–1.2

## Data Availability

The authors declare that the data supporting the findings of this study are available within the article. Additional information is available from the authors upon reasonable request.

## Abbreviations

The following abbreviations are used in this manuscript:

CCHFV	Crimean-Congo hemorrhagic fever virus
DAS-ELISA	Double-Antigen Sandwich Enzyme-Linked Immunosorbent Assay
q RT-PCR	quantitative reverse-transcription polymerase chain reaction
CROOH	Establishment of a coordinated disease surveillance system (in Croatia) in accordance with the OH approach
